# Human papillomavirus and survival of patients per histological subsite of tonsillar squamous cell carcinoma

**DOI:** 10.1002/cam4.1400

**Published:** 2018-03-23

**Authors:** Linnea Haeggblom, Tove Attoff, Lalle Hammarstedt‐Nordenvall, Anders Näsman

**Affiliations:** ^1^ Department of Oncology‐Pathology Karolinska Institutet 171 76 Stockholm Sweden; ^2^ Department of Oto‐Rhino‐Laryngology, Head and Neck Surgery Karolinska University Hospital 171 76 Stockholm Sweden; ^3^ Department of Clinical Science, Intervention and Technology Karolinska Institutet 171 76 Stockholm Sweden; ^4^ Department of Clinical Pathology Karolinska University Hospital 171 76 Stockholm Sweden

**Keywords:** Human papillomavirus, oropharyngeal cancer, survival, tonsillar cancer

## Abstract

Current data advocate that oropharyngeal squamous cell carcinoma (OPSCC) should be divided into subsites when evaluating the presence of human papillomavirus (HPV) and prognosis. More specifically, tonsillar squamous cell carcinoma (TSCC) and base of tongue squamous cell carcinoma (BOTSCC) have much higher HPV prevalence compared to other OPSCC. Moreover, patients with HPV positive (HPV+) TSCC and BOTSCC have a better prognosis as compared to patients with HPV negative (HPV−) corresponding tumors, while the prognostic role of HPV in other OPSCC is unclear. Furthermore, in a recent report from Denmark, TSCC was further subclassified into specified TSCC (STSCC) and nonspecified TSCC (NSTSCC), with HPV significantly more prevalent in STSCC. In this study, the histopathological influence of HPV prevalence and survival in TSCC was analyzed in a TSCC cohort with known HPV status, of patients diagnosed 1970–2002 in Stockholm. In total, 139 TSCC biopsies with both tumor and adjacent normal tissue were separated into STSCC and NSTSCC. HPV was significantly more commonly found in STSCC than in NSTSCC. Patients with HPV+ STSCC had a better disease‐specific and overall survival as compared to patients with HPV+ NSTSCC, but no survival differences were observed in patients with HPV− STSCC and NSTCC. These findings confirm previous reports and suggest that TSCC subsite may also be of relevance for clinical outcome and should be further followed up in future studies.

## Introduction

It is only a decade ago since the International Agency of Research on Cancer (IARC) declared a casual role of human papillomavirus (HPV) in the etiology of cancer of the oropharynx and the tonsil 1. Since then, HPV‐positive (HPV+) and HPV‐negative (HPV−) oropharyngeal squamous cell carcinoma (OPSCC) have been accepted as two different diseases, with a more favorable outcome in the former as compared to the latter. Consequently, according to the latest American Joint Committee on Cancer (AJCC) staging manual, 8th edition, OPSCC should be separated into HPV‐associated and nonassociated tumors, as classified by p16INK4a (p16) overexpression by immunohistochemistry (IHC) 2, 3. However, accumulating data suggests that OPSCC should be divided into subsites when assessing HPV and outcome. In a recent published meta‐analysis, the HPV prevalence varied considerably between different OPSCC subsites 4. While tumors arising in tissues harboring crypts (TSCC and BOTSCC) had high HPV numbers, the tumors at the other subsites (cancer of the soft palate and oropharyngeal walls) had lower HPV prevalence, similar to that in oral cancer 4. Furthermore, recent data also imply that the prognostic impact of HPV differs between these subsites 5, 6, 7, 8, 9, 10, 11, 12, 13. Moreover, the tonsils are lined with two different epithelial structures: (1) an epithelium covering the tonsil with similarities to that in the oral cavity and (2) an epithelium that invaginates and forms crypts and there is evidence for HPV+ carcinomas developing from these crypts 14, 15. Therefore, Garnaes et al. subclassified TSCC into specified TSCC (STSCC) and nonspecified TSCC (NSTCC), with HPV significantly more prevalent in STSCC than in NSTSCC and with a more pronounced correlation between survival and HPV in the latter 7, 10. Here, we aimed to examine the histopathological impact on HPV status prevalence and survival in TSCC in a separate cohort of TSCC patients diagnosed 1970–2002 in Stockholm.

## Material and Methods

### Patients and histomorphological classification

In total, 203 patients diagnosed with TSCC (ICD‐7 145.0) between 1970 and 2002 together with clinical data and HPV status (presence of HPV DNA tested with PCR) were obtained from previous publications 8, 16. The diagnosis of TSCC was based on clinical, radiological, and pathological data. Available hematoxylin‐eosin (H&E) slides from the tumors were evaluated by a pathologist blinded for clinical and molecular data. Available tumors with representation of normal surrounding tissue (*n* = 139) were separated into STSCC (presence of tonsillar crypts and tonsillar lymphoid tissue with germinal centers) and NSTSCC (absence of tonsillar crypts and tonsillar lymphoid tissue with germinal centers) based on the histomorphological context according to Garnaes et al.*,* with the exception of including only tumor specimens containing both tumor tissue and normal tissue 7, 10. Patient and tumor characteristics of the study cohort as well as the total cohort are depicted in Tables 1 and S1. The study was approved by the Ethical Committee at Karolinska Institutet according to ethical permission 2005/431‐31/4.

**Table 1:** Available tumors and their characteristics, obtained from previous publications 8, 16

Tumor and patient characteristics	Number of patients			*P*‐value
	HPV+ (*N* = 75)	HPV− (*N* = 64)	All (*N* = 139)	
Age at diagnosis (mean, years)	56.3	65.1	60.4	<0.001^a^
Sex				
Male	51 (55%)	41 (45%)	92	0.6^b^
Female	24 (51%)	23 (49%)	47	
TNM stage^c^				
I	0 (0%)	2 (100%)	2	0.04^a^
II	7 (64%)	4 (36%)	11	
III	22 (69%)	10 (31%)	32	
IV	37 (55%)	30 (45%)	67	
Unknown	9 (33%)	18 (67%)	27	
Histopathology grade				
High	7 (50%)	7 (50%)	14	0.06^a^
Moderate	26 (43%)	34 (57%)	60	
Low	42 (65%)	23 (35%)	65	
Treatment				
Preoperative radiotherapy^d^	48 (71%)	20 (29%)	68	<0.001^a^
Postoperative radiotherapy^d^	8 (67%)	4 (33%)	12	
Radiotherapy only^d^	12 (44%)	15 (56%)	27	
Surgery only	1 (100%)	0 (0%)	1	
Palliative treatment	6 (19%)	25 (81%)	31	

a Chi‐square test.

b Independent student *t*‐test.

c TNM stage according to UICC 1997.

d Conventional radiotherapy (2.0 Gy/day, total dose: 68 Gy).

### Statistical analysis

Statistical analyses were performed in STATA (v11.1 StataCorp, College Station, TX) and SPSS (IBM SPSS Statistics for Mac, Version 20.0. Armonk, NY: IBM Corp. USA). The Chi‐square test was used to compare categorical data. Survival was measured in months from the date of diagnosis until a defined event or until 5 years from diagnosis. An event was defined as death of any cause (overall survival) or death with disease (disease‐specific survival). Patients who died without a documented TSCC were censored at the death date in DSS. All patients were censored after 5 years (60 months). Only patients treated with the intent to cure were included in the survival analysis, as previously described 8. Survival was estimated by means of the Kaplan–Meier method and was compared between the two groups (STSCC and NSTSCC) with the use of the log‐rank test. The Cox proportional hazard model was used to calculate the unadjusted and adjusted hazard ratios (HR).

## Results

**Figure 1:**
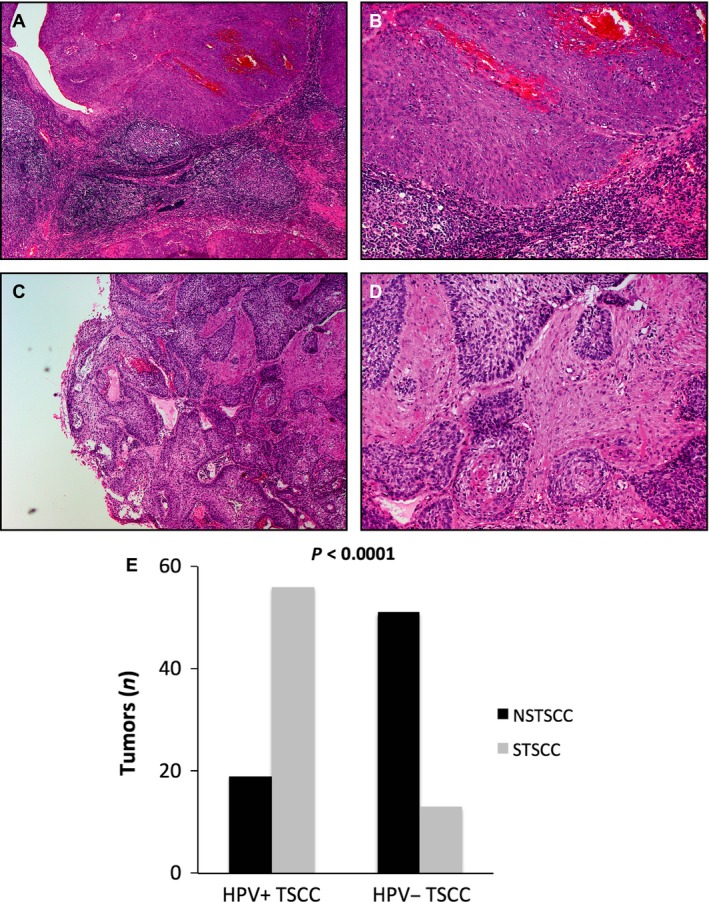
Representative hematoxylin‐eosin stainings of specified tonsillar squamous cell carcinoma (STSCC) and nonspecified tonsillar squamous cell carcinoma (NSTSCC). (A) 4x STSCC. (B) 10x STSCC. (C) 4x NSTSCC. (D) 10x NSTSCC. (E) Histomorphological subgrouping STSCC/NSTSCC of tonsillar squamous cell carcinoma in relation to human papillomavirus (HPV) status (estimated as presence of HPV DNA).

In total, 139 of the analyzed TSCC samples had both tumor and adjacent normal tissue and could therefore be separated into STSCC (Fig. 1A and B) and NSTSCC (Fig. 1C and D). Of all 139 TSCC samples analyzed 50% were STSCC and 50% NSTSCC. There was a significant overrepresentation of STSCC in patients with HPV+ TSCC as compared to patients with HPV− TSCC (56 (75%) HPV+ STSCC and 19 (25%) HPV+ NSTSCC vs. 13 (20%) HPV− STSCC and 51 (80%) HPV− NSTSCC; *P* < 0.0001) (Fig. 1E). Neither tumor stage nor tumor differentiation was correlated with histomorphological subclassification (data not shown).

**Figure 2:**
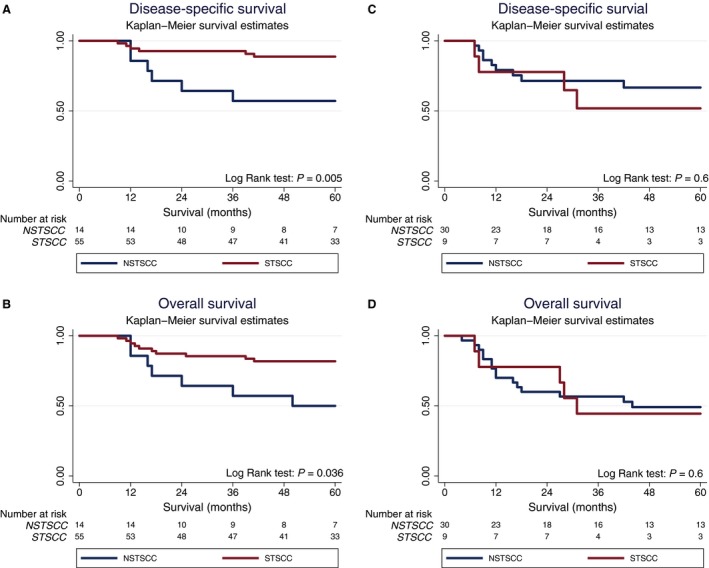
Kaplan–Meier curves for disease‐specific survival (DSS) and overall survival (OS) in patients with human papillomavirus positive (HPV+) and negative (HPV−) tonsillar squamous cell carcinoma (TSCC) per histomorphological subgroup, *that is,* specified and nonspecified TSCC (STSCC and NSTSCC, respectively). (A) DSS stratified for STSCC/NSTSCC in patients with HPV+ TSCC, (B) OS stratified for STSCC/NSTSCC in patients with HPV+ TSCC, (C) DSS stratified for STSCC/NSTSCC in patients with HPV− TSCC, (D) OS stratified for STSCC/NSTSCC in patients with HPV− TSCC.

Moreover, patients with HPV+ STCC had a significantly better 5‐year DSS and OS as compared to patients with HPV+ NSTSCC (Fig. 2A and B). More specifically, in a Kaplan–Meier analysis, patients with HPV+ STSCC had better OS and DSS than patients with HPV+ NSTSCC (*P* = 0.005 and *P* = 0.036 for DSS and OS, respectively, by the log‐rank test). In a multivariate analysis including tumor stage, tumor differentiation and patient age upon diagnosis, histomorphological subclassification was still correlated with survival in patients with HPV+ TSCC. The unadjusted and adjusted HR for DSS were 4.4 (95% confidence interval, CI: 1.4–14, *P* = 0.01) and 7.3 (95% CI: 1.7–30.8, *P* = 0.007), respectively. Corresponding HR for OS were 2.3 (95% CI: 1.0–5.7, *P* = 0.04) and 1.9 (95% CI: 0.71–5.1, *P* = 0.2), respectively.

No significant differences in survival between STCC and NSTSCC were observed in patients with HPV− TSCC (Fig. 2C and D). The 5‐year DSS was 56% (95% CI: 23–85%) for HPV− STCC and 67% (95% CI: 48–83%) for HPV− NSTSCC, while the OS was 44% (95% CI: 15–77%) for HPV− STCC and 46% (95% CI: 28–66%) for HPV− NSTSCC (Fig. 2C and D). No correlation with survival was observed in the multivariable analysis (data not shown).

## Discussion

In this brief report, HPV+ status was significantly more frequently associated with STSCC as compared to NSTSCC, and moreover, patients with HPV+ STSCC had a significantly better prognosis compared to those with HPV+ NSTSCC. In contrast, HPV− status was significantly more often found in NSTSCC, but the histomorphological subtype of HPV− TSCC (NSTSCC or STSCC) did not affect patients’ prognosis.

The relation of HPV status and TSCC subsite was clearly in agreement with the study by Garnaes et al. where a similar relationship was described in a Danish cohort 7, 10. During recent years, the focus of HPV in head and neck cancer has shifted toward studying HPV mainly in OPSCC 17. However, accumulating data suggests that also the concept of HPV in OPSCC is too broad and unspecific, especially in relation to when HPV status is utilized as a marker for treatment and clinical outcome 4, 5, 6, 7, 10. The present data are in line with such assumptions and support the notion that HPV infection is highly associated with “lymphoepithelial” tissues.

Interestingly, patients with HPV+ STSCC had a better DSS and OS as compared to HPV+ NSTSCC. These data are also in line with previous data, in which HPV only had a prognostic impact in tumors arising in tissues harboring crypts 5, 6, 8, 9, 10.

There are recognizable limitations in this study. A major limitation was that for this cohort we only had access to hematoxylin‐eosin stained slides; moreover, no tumor material was available for p16 IHC staining. However, in cohorts partly overlapping with the present one, we have reported that p16 overexpression was highly concordant and significantly associated with HPV+ DNA status 18, 19. Here, we assume a similar correlation. Another limitation is that the biopsies/resections may not give the full picture of the entire tumor and that although samples missing either tumor or normal tissue were excluded, the actual location of the biopsy may in some cases have influenced the histomorphological classification. Lastly, the study design was retrospective and patients were obtained from a previous nonrandomized study 8, 16. Included patients in this study did not differ from excluded patients, with the exception of that excluded patients were significantly more often male and had HPV negative tumors (Table S1). This was due to the fact that most excluded tumors were from patients diagnosed between 1970 and 1980 (data not shown), when HPV prevalence was much lower 16.

Taking these limitations into account, our data still support the data of Garnaes et al. 7, 10, and thus subclassification into STSCC and NSTSCC may confer further precision when deciding which patients with HPV+ cancer can potentially be offered less intensive therapy. Nonetheless, there is a need for additional studies evaluating this newly presented subclassification in relation to HPV status together with clinical outcome.

In conclusion, the present study shows that HPV+ status is mainly observed in STSCC as compared to NSTSCC and vice versa. Moreover, HPV+ status only had a positive survival value in the context of STSCC and not in NSTSCC. Together these data suggest that the context of the tumor is essential for evaluating HPV prevalence and tumor prognostication and that the concept of OPSCC is too broad when separating patients with HPV+ and HPV− tumours.

## Conflict of Interest

No conflict of interest to declare.

## Supporting information


**Table S1.** Total patient cohort with included and excluded cases and their characteristics, obtained from previous publications 8, 16.
