# Supplementary material for: Human papillomavirus and survival of patients per histological subsite of tonsillar squamous cell carcinoma
Source: Cancer Med. 2018 Mar 23;7(5):1717–22. doi: 10.1002/cam4.1400 (PMC5943436; doi:10.1002/cam4.1400)
Supplement: Supplementary file 1 — Table S1. Total patient cohort with included and excluded cases and their characteristics, obtained from previous publications 8, 16. [file CAM4-7-1717-s001.docx]

**Supplementary Table.** Total patient cohort with included and excluded cases and their characteristics, obtained from previous publications [8, 16].

| **Tumour and patient characteristics** | **Number of patients** | | |  |
| --- | --- | --- | --- | --- |
|  | *Included* | *Excluded* | *All* |  |
|  | (N=139) | (N=64) | (N=203) | p-value |
| Age at diagnosis (mean, years) | 60.4 | 63.0 | 61.5 | 0.1 ^¶^ |
| *Sex* |  |  |  | 0.02^#^ |
| Male | 92 (63%) | 53 (37%) | 145 |  |
| Female | 47 (81%) | 11 (19%) | 58 |  |
| *TNM stage*^*^ |  |  |  | 0.7^#^ |
| I | 2 (100%) | 0 (0%) | 2 |  |
| II | 11 (73%) | 4 (27%) | 15 |  |
| III | 32 (63%) | 19 (37%) | 51 |  |
| IV | 67 (71%) | 28 (29%) | 95 |  |
| Unknown | 27 (68%) | 13 (32%) | 40 |  |
| *Histopathology grade* |  |  |  | 0.2^#^ |
| High | 14 (%) | 7 (%) | 21 |  |
| Moderate | 60 (%) | 25 (%) | 85 |  |
| Low | 65 (%) | 30 (%) | 95 |  |
| Unknown | 0 (0%) | 2 (%) | 2 |  |
| *Treatment* |  |  |  | 0.1^#^ |
| Preoperative radiotherapy*^§^* | 68 (78%) | 19 (22%) | 87 |  |
| Postoperative radiotherapy*^§^* | 12 (71%) | 5 (29%) | 17 |  |
| Radiotherapy only*^§^* | 27 (59%) | 19 (41%) | 46 |  |
| Surgery only | 1 (50%) | 1 (50%) | 2 |  |
| Palliative treatment | 31 (61%) | 20 (39%) | 51 |  |
| *HPV DNA status* |  |  |  | 0.03^#^ |
| Positive | 75 (76%) | 24 (24%) | 99 |  |
| Negative | 64 (62%) | 40 (38%) | 104 |  |

^*^ TNM stage according to UICC 1997.

^§^ Conventional radiotherapy (2.0 Gy/day, total dose: 68 Gy).

^#^ Chi^2^ test

^¶^ Independent student t-test
